# Sero-prevalence and risk factors for Severe Acute Respiratory Syndrome Coronavirus 2 infection in women and children in a rural district of Bangladesh: A cohort study

**DOI:** 10.7189/jogh.12.05030

**Published:** 2022-07-23

**Authors:** Rasheda Khanam, Shafiqul Islam, Sayedur Rahman, Salahuddin Ahmed, Ashraful Islam, Tarik Hasan, Emran Hasan, Nabidul Haque Chowdhury, Arunangshu Dutta Roy, Iffat Ara Jaben, Asim A Nehal, Sachiyo Yoshida, Alexander A Manu, Rubhana Raqib, Eric D McCollum, Mohammod Shahidullah, Fyezah Jehan, Sunil Sazawal, Rajiv Bahl, Abdullah H Baqui

**Affiliations:** 1 Department of International Health, Johns Hopkins Bloomberg School of Public Health, Baltimore, Maryland, USA; 2 Projahnmo Research Foundation, Dhaka, Bangladesh; 3 Department for Maternal, Child, Adolescents and Ageing Health, World Health Organization, Geneva, Switzerland; 4 Department of Epidemiology and Disease Control, University of Ghana School of Public Health, Legon, Accra, Ghana; 5 International Center for Diarrheal Disease Research, Bangladesh, Mohakhali, Dhaka, Bangladesh; 6 Global Program for Pediatric Respiratory Sciences, Eudowood Division of Paediatric Respiratory Sciences, School of Medicine, Johns Hopkins University, Baltimore, Maryland, USA; 7 Bangabandhu Shaikh Mujib Medical University, Dhaka, Bangladesh; ^8^Department of Paediatrics and Child Health, Aga Khan University, Karachi, Sind Pakistan ^9^Center for Public Health Kinetics, Global Division, LGL Vinoba Puri, Lajpat Nagar II, New Delhi, India; 10 Public Health Laboratory-IDC, Chake Chake, Pemba, Tanzania.

## Abstract

**Background:**

Bangladesh reported its first COVID-19 case on March 8, 2020. Despite lockdowns and promoting behavioural interventions, as of December 31, 2021, Bangladesh reported 1.5 million confirmed cases and 27 904 COVID-19-related deaths. To understand the course of the pandemic and identify risk factors for SARs-Cov-2 infection, we conducted a cohort study from November 2020 to December 2021 in rural Bangladesh.

**Methods:**

After obtaining informed consent and collecting baseline data on COVID-19 knowledge, comorbidities, socioeconomic status, and lifestyle, we collected data on COVID-like illness and care-seeking weekly for 54 weeks for women (n = 2683) and their children (n = 2433). Between March and July 2021, we tested all participants for SARS-CoV-2 antibodies using ROCHE's Elecsys® test kit. We calculated seropositivity rates and 95% confidence intervals (95% CI) separately for women and children. In addition, we calculated unadjusted and adjusted relative risk (RR) and 95% CI of seropositivity for different age and risk groups using log-binomial regression models.

**Results:**

Overall, about one-third of women (35.8%, 95% CI = 33.7-37.9) and one-fifth of children (21.3%, 95% CI = 19.2-23.6) were seropositive for SARS-CoV-2 antibodies. The seroprevalence rate doubled for women and tripled for children between March 2021 and July 2021. Compared to women and children with the highest household wealth (HHW) tertile, both women and children from poorer households had a lower risk of infection (RR, 95% CI for lowest HHW tertile women (0.83 (0.71-0.97)) and children (0.75 (0.57-0.98)). Most infections were asymptomatic or mild. In addition, the risk of infection among women was higher if she reported chewing tobacco (RR = 1.19,95% CI = 1.03-1.38) and if her husband had an occupation requiring him to work indoors (RR = 1.16,  95% CI = 1.02-1.32). The risk of infection was higher among children if paternal education was >5 years (RR = 1.37, 95% CI = 1.10-1.71) than in children with a paternal education of ≤5 years.

**Conclusions:**

We provided prospectively collected population-based data, which could contribute to designing feasible strategies against COVID-19 tailored to high-risk groups. The most feasible strategy may be promoting preventive care practices; however, collecting data on reported practices is inadequate. More in-depth understanding of the factors related to adoption and adherence to the practices is essential.

The Severe Acute Respiratory Syndrome Coronavirus 2 (SARS-CoV-2) or COVID-19 pandemic has created an unprecedented public health crisis [1]. Although we have been experiencing the pandemic for about two years, the infections are still rising worldwide. The emergence of multiple variants resulted in about 300 million confirmed cases and approximately 5.5 million deaths as of December 31, 2021 [2]. Most symptomatic patients with COVID-19 present with flu-like symptoms, primarily cough, fever, and headache. Common complications include pneumonia, acute respiratory distress syndrome, septic shock, and cardiovascular manifestations [3]. Several public health measures, including hand hygiene, social distancing, and face masks, have been the primary means of limiting disease spread and saving lives [3-5].

Bangladesh reported its first case of COVID-19 on March 8, 2020. Between March 26, 2020, and August 11, 2021, the government of Bangladesh (GoB) imposed several rounds of lockdown in the country, enforced many public health measures, including physical distancing and wearing of masks, and promoted behavioural intervention, including washing hands. Despite all these measures, Bangladesh experienced three epidemic waves: the first from April to September 2020, the second from March to October 2021, and the third from January to March 2022, approximately. As of December 31, 2021, Bangladesh reported 1.5 million confirmed cases of COVID-19 and 27 904 deaths [6]. These numbers are likely to be underestimated because of inadequate testing facilities in Bangladesh [7-9].

Epidemiological studies to measure the true extent of SARS-CoV-2 infection are vital for pandemic projections and planning appropriate pandemic containment strategies. Most SARS-CoV-2 infections are asymptomatic or mild [10,11] and are unlikely to be identified in routine surveillance, particularly in populations characterized by low care-seeking and limited availability of testing. Population-based serosurvey is an essential tool to understand the trajectories of the pandemic. It helps estimate the proportion of the population previously infected with SARS-CoV-2, its spread in the communities, and the proportion of the population still susceptible to infection. In addition, it generates crucial intelligence on the rate of asymptomatic infections.

Another critical use of serosurveys is identifying risk factors for infection and disease severity in different population groups. For example, poor living conditions, crowding, older age, and comorbidities including obesity, diabetes, and hypertension were shown to increase the risk of COVID-19 infection and severity [12]. A better understanding of the risk factors would facilitate targeting prevention and control measures to reduce transmission and fine-tuning management strategies to minimize severe outcomes.

We conducted this study to estimate the proportion of the population infected with SARS-CoV-2 and identify its risk factors in a cohort of women and their children in the rural Sylhet district of Bangladesh.

## METHODS

### Study design, participants, and settings

We enrolled and prospectively followed all mothers and their children participating in the Bangladesh site of the multi-country Alliance for Maternal and Newborn Health Improvement (AMANHI) biorepository study. The site is located in northeast Bangladesh's Zakiganj Upazila of Sylhet District. The detailed methodology of the AMANHI study is published elsewhere [13]. This AMANHI-COVID-19 study was conducted between November 16, 2020, and December 31, 2021. After taking informed consent, baseline participant data on COVID-19 knowledge, comorbidity, socioeconomic status, and lifestyle were collected. In addition, weekly morbidity surveillance to collect data separately on women and children on COVID-like illness (CLI), care-seeking, and hospitalization were instituted. Local community health workers (CHWs) with a minimum of a 10th-grade education and six weeks of training on study tools and procedures collected the data. Data were collected via cell phone in the first six weeks due to local pandemic restrictions. Our project database contained the cell phone number of about 80% of the women. From January 1, 2021, study staff made household visits.

A blood sample for the SARs-CoV-2 antibody test was collected from all consenting mothers and their children between March 1 and July 31, 2021. For the convenience of study participants, blood samples were obtained in three locations: community clinics, homes, and the study laboratory at Zakiganj Upazila health complex. After obtaining written consent, trained phlebotomists collected 2-3 ml of venous blood from each study participant. The sample was transported to the study laboratory for centrifugation, serum separation, and storage at -80°C. The serum aliquots were processed in a BSL2 bio-safety cabinet per international guidelines [14] for handling COVID-19 blood samples. One aliquot of serum sample ( ~ 300 μL) from each participant was sent to the International Centre for Diarrhoeal Disease Research Bangladesh (icddr, b) laboratory in Dhaka, and the remaining aliquots were stored at -80°C.

We used ROCHE's Elecsys®Anti-SARS-CoV-2 antibody test. The Elecsys® is an immunoassay for the in vitro qualitative detection of antibodies to SARS-CoV-2 infection in human serum and plasma. Since this test is 99.5% sensitive and 99.8% specific and was used in a population with almost no exposure to the COVID-19 vaccine, the antibody positivity was considered a marker of past or recent symptomatic or asymptomatic COVID-19 infections.

### Measurements and data analysis

Seropositivity rates and 95% confidence intervals were calculated separately for women and children and by different age and risk groups. We examined the association of the following epidemiologically relevant risk factors data: individual, household, socioeconomic characteristics, lifestyle, and reported preventive care practices with COVID-19 infection. The characteristics of women included age (<30 years and ≥30 years), education (0-5 years and above 5 years of schooling), occupation (housewife, working/employed), body mass index (BMI, underweight and normal <25 and obese/overweight ≥25), and the presence of comorbidities. The BMI was calculated as the ratio of weight in kilograms to height in meters squared (kg/m^2^) [15]. The household wealth index was constructed using a standard principal component analysis (PCA) approach that incorporated household type and possessions data and were divided into tertiles (ie, low, medium, high) [16]. We categorized data on husbands' occupations into two distinct groups based on the nature and place of the job. Those who worked for the government, the private sector, or were self-employed were grouped and labelled as indoor workers. Others who reported working as day labourers, farmers, or unemployed were classified as outdoor workers. We used the Hot deck method to impute the missing data.

We conducted a similar analysis of child-level data. We created a variable called COVID Like Illness (CLI) if a woman or a child reported one or more of these symptoms: 1) acute onset of fever and cough or 2) acute onset of any three or more of the following signs or symptoms: fever, cough, general weakness/fatigue, headache, myalgia, sore throat, coryza, dyspnoea, anorexia/nausea/vomiting, diarrhoea, altered mental status or 3) recent onset of anosmia/loss of smell or ageusia/loss of taste in the absence of any other identified cause [17]. We then calculated the proportion of women and children who had at least one episode of CLI in the 3-month and 6-month preceding the antibody test. We examined the prevalence of four preventive care practices: staying at home as much as possible, physical distancing when outside the household, use of mask and hand washing, and their association with COVID antibody test positivity in women and children. We calculated unadjusted and adjusted relative risk and 95% confidence intervals (RR, 95% CI) of seropositivity using log-binomial regression models. We included covariates in bivariable models with a *P-*value of <0.2 in the adjusted models.

### Ethical approval and consent process

We obtained ethical approval from the Johns Hopkins University Institutional Review Board (IRB00012923), the Ethical Review Committee of icddr, b, (PR-20055), and the Ethical Review Committee of the World Health Organization (CERC. 0006). The consent procedure was 2-folds. First, verbal consent was obtained for baseline and morbidity data collection by CHWs after explaining the purpose of the study to the women. We obtained separate written consent for the collection of blood. Study team members were trained in safety measures, including physical distancing, mask use, and hand hygiene, and study participants were provided with a mask for the participants' safety. The phlebotomist received additional training in personal protective equipment and the safe transfer of biological materials to the study laboratory.

## RESULTS

**Figure 1:**
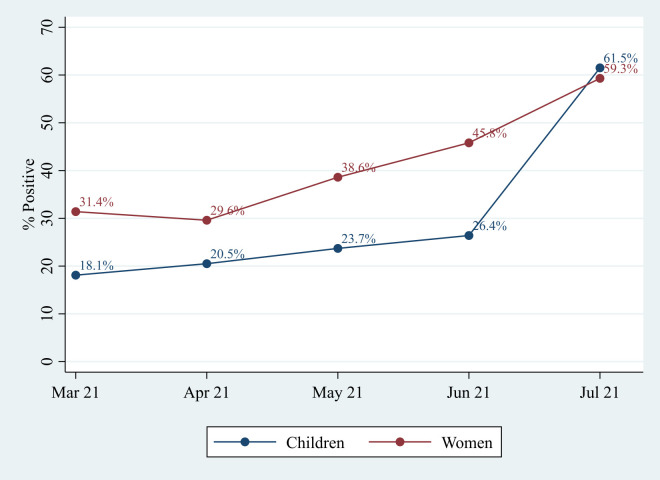
The proportion of women and children positive for COVID-19 antibody by study month.

We collected blood samples from 2041 (76.1%) of the 2683 women in the cohort. We could not obtain a sample from 642 women for the following reasons: 69 (2.6%) migrated out, 53 (2.0%) were absent after repeated attempts, 518 (19.3%) refused the blood draw, and phlebotomists failed blood collection in two women. Similarly, we collected blood samples from 1398 (57.5%) of the 2433 children in the cohort. Reasons for not collecting the samples included: 67 migrated out (2.8%), 57 absent (2.3%), 902 (37.1%) refused to donate blood, blood could not be collected from eight children, and one child died. Seven hundred and thirty women (35.8%, 95% CI = 33.7-37.9) had detectable SARs-CoV-2 antibodies, while 298 children (21.3%, 95 CI = 19.2-23.6) were positive for SARs-Cov-2 antibodies. About a third of the women and about one-fifth of the children tested during March-April 2021 were antibody positive. The positivity rate increased slightly in May and rapidly increased in June 2021. By July 2021, about 60% of the mothers and their children were antibody positive (**Figure 1**).

In an adjusted model, among women, three variables, chewing tobacco, husband's occupation, and household wealth status, were associated with antibody positivity (**Table 1**). Women with husbands' occupations requiring indoor work had a 16% higher risk (RR = 1.16, 95% CI = 1.02-1.32) than women whose husbands were outdoor workers. Women from low and medium wealth tertiles had a 17% lower risk (RR = 0.83, 95% CI = 0.71-0.97) than women in a high-wealth group. Children with paternal education of 0-5 years had a 37% higher risk (RR = 1.37, 95% CI = 1.10-1.71) antibody positivity than children whose paternal education was >5 years. Compared to children from high household wealth, antibody positivity among children from households with low- and medium- wealth tertiles was 25% (RR = 0.75, 95% CI = 0.57-0.98) and 32% (RR = 0.68, 95% CI = 0.52-0.89) lower, respectively (**Table 2**).

**Table 1:** Association of selected characteristics of women and households with COVID-19 antibody test positivity

Selected Characteristics	Total N = 2041	Positive n (%)	Negative n (%)	*P-*value	Unadjusted RR (95% CI)	Adjusted RR (95% CI)
***Women Characteristics***						
**Age in years**						
<30	1361	481 (35.3)	880 (64.7)		Ref	
≥30	680	249 (36.6)	431 (63.4)	0.57	1.04 (0.92-1.17)	
**Education**						
0-5 y	898	298 (33.2)	600 (66.8)		Ref	Ref
>5 y	1143	432 (37.8)	711 (62.2)	0.03	1.14 (1.01-1.28)	1.03 (0.90-1.17)
**Occupation**						
Housewife	2012	718 (35.7)	1294 (64.3)		Ref	
Working/employed	29	12 (41.4)	17 (58.6)	0.53	1.16 (0.75-1.80)	
**BMI**						
Thin and normal (BMI<25 kg/m^2^)	1920	678 (35.3)	1242 (64.7)		Ref	Ref
Overweight/Obese (BMI≥25 kg/m^2^)	121	52 (43.0)	69 (57.0)	0.09	1.22 (0.98-1.51)	1.12 (0.90-1.39)
**Chewing tobacco**						
Yes	357	140 (39.2)	217 (60.8)		1.12 (0.97-1.29)	1.19 (1.03-1.38)
No	1684	590 (35.0)	1094 (65.0)	0.13	Ref	Ref
Husband's education*						
0-5 y	1395	475 (34.1)	920 (65.9)		Ref	Ref
>5 y	640	253 (39.5)	387 (60.5)	0.02	1.16 (1.03-1.31)	1.05 (0.92-1.20)
Husband's occupation*						
Govt/private/self-employed (possibly in-door)	744	305 (41.0)	439 (59.0)		1.25 (1.11-1.41)	1.16 (1.02-1.32)
Daily wage/farming/other/does not work (possibly out-door)	1291	423 (32.8)	868 (67.2)	<0.01	Ref	Ref
***Prompted preventive measures***						
**Staying at home as much as possible**						
Yes	1985	707 (35.6)	1278 (64.4)		Ref	
No	56	23 (41.1)	33 (58.9)	0.40	1.15 (0.84-1.59)	
**Physical distancing with other persons outside the household**						
Yes	1975	713 (36.1)	1262 (63.9)		Ref	Ref
No	66	17 (25.8)	49 (74.2)	0.09	0.71 (0.47-1.08)	0.74 (0.49-1.12)
**Cleaning hands regularly with soap and water or hand rub**						
Yes	2027	723 (35.7)	1304 (64.3)		Ref	
No	14	7 (50.0)	7 (50.0)	0.27	1.40 (0.83-2.37)	
**Wearing a face mask when around others**						
Yes	1983	711 (35.9)	1272 (64.1)		Ref	
No	58	19 (32.8)	39 (67.2)	0.63	0.91 (0.63-1.33)	
***Household characteristics***						
**Number of adults in the household**						
≤2	705	235 (33.3)	470 (66.7)		Ref	Ref
>2	1336	495 (37.1)	841 (62.9)	0.10	1.11 (0.98-1.26)	1.09 (0.96-1.23)
**Number of living children in the household**						
≤2	1338	474 (35.4)	864 (64.6)		Ref	
>2	703	256 (36.4)	447 (63.6)	0.66	1.03 (0.91-1.16)	
**Wealth index (tertiles)**						
Poor	774	247 (31.9)	527 (68.1)		0.74 (0.65-0.85)	0.83 (0.71-0.97)
Middle	634	211 (33.3)	423 (66.7)	<0.01	0.77 (0.67-0.89)	0.82 (0.71-0.96)
Rich	633	272 (43.0)	361 (57.0)		Ref	Ref

CI – confidence interval, RR – relative risk, Ref – reference

*Husbands of 6 women died before the beginning of the study.

**Table 2:** Association of selected characteristics of children and their household with COVID-19 antibody test positivity

Selected Characteristics	Total N = 1398	Positive n (%)	Negative n (%)	*P-* value	Unadjusted RR (95% CI)	Adjusted RR (95% CI)
***Children Characteristics***						
**Gender**						
Male	673	137 (20.4)	536 (79.6)		Ref	
Female	725	161 (22.2)	564 (77.8)	0.40	1.09 (0.89-1.34)	
**Age in months**						
35-59 mo	842	186 (22.1)	656 (77.9)		1.10 (0.89-1.35)	
≥60 mo	556	112 (20.1)	444 (79.9)	0.38	Ref	
**Stunting**						
Normal (-1.99, 6)	648	151 (23.3)	497 (76.7)		Ref	Ref
Stunting (-2.99,-2)	487	105 (21.6)	382 (78.4)	<0.05	0.93 (0.74-1.15)	1.00 (0.80-1.24)
Severe Stunting (≤-3)	263	42 (16.0)	221 (84.0)		0.69 (0.50-0.93)	0.81 (0.59-1.11)
**Wasting**						
No	1320	279 (21.1)	1041 (78.9)		Ref	
Yes (Weight-for-height<-2)	78	19 (24.4)	59 (75.6)	0.50	1.15 (0.77-1.73)	
**Underweight**						
Normal (-1.99, 6)	871	188 (21.6)	683 (78.4)		Ref	
Underweight (-2.99, -2)	403	86 (21.3)	317 (78.7)	0.85	0.99 (0.79-1.24)	
Severe underweight (≤-3)	124	24 (19.4)	100 (80.6)		0.90 (0.61-1.31)	
***Maternal Characteristics***						
Mother's age in years*						
<30	927	200 (21.6)	727 (78.4)		Ref	
≥30	464	97 (20.9)	367 (79.1)	0.77	0.97 (0.78-1.20)	
Mother's education*						
0-5 y	602	114 (18.9)	488 (81.1)		Ref	Ref
>5 y	789	183 (23.2)	606 (76.8)	0.06	1.22 (0.99-1.51)	0.95 (0.75-1.21)
Mother's occupation*						
Housewife	1372	291 (21.2)	1081 (78.8)		Ref	
Working/employed	19	6 (31.6)	13 (68.4)	0.27	1.49 (0.76-2.91)	
Father's education**						
0-5 y	928	167 (18.0)	761 (82.0)		Ref	Ref
>5 y	467	130 (27.8)	337 (72.2)	<.01	1.55 (1.27-1.89)	1.37 (1.10-1.71)
Father's occupation**						
Govt/private/self-employed (possibly in-door)	534	129 (24.2)	405 (75.8)		1.24 (1.01-1.52)	1.00 (0.80-1.24)
Daily wage/farming/other/does not work (possibly out-door)	861	168 (19.5)	693 (80.5)	0.04	Ref	Ref
***Prompted preventive measures***						
**Staying at home as much as possible**						
Yes	1364	292 (21.4)	1072 (78.6)		Ref	
No	34	6 (17.6)	28 (82.4)	0.60	0.82 (0.40-1.72)	
**Physical distancing with other persons outside the household**						
Yes	1358	292 (21.5)	1066 (78.5)		Ref	
No	40	6 (15.0)	34 (85.0)	0.32	0.70 (0.33-1.47)	
**Cleaning hands regularly with soap and water or hand rub**						
Yes	1388	295 (21.3)	1093 (78.7)		Ref	
No	10	3 (30.0)	7 (70.0)	0.45	1.41 (0.54-3.66)	
**Wearing a face mask when around others**						
Yes	1359	290 (21.3)	1069 (78.7)		Ref	
No	39	8 (20.5)	31 (79.5)	0.90	0.96 (0.51-1.80)	
***Household Characteristics***						
**Number of adults in the household**						
≤2	483	90 (18.6)	393 (81.4)		Ref	Ref
>2	915	208 (22.7)	707 (77.3)	0.08	1.22 (0.98-1.52)	1.10 (0.87-1.40)
**Number of living children in the household**						
≤2	868	197 (22.7)	671 (77.3)		Ref	Ref
>2	530	101 (19.1)	429 (80.9)	0.11	0.84 (0.68-1.04)	0.90 (0.71-1.14)
**Wealth Index (tertiles)**						
Poor	513	92 (17.9)	421 (82.1)		0.63 (0.50-0.80)	0.75 (0.57-0.98)
Middle	420	74 (17.6)	346 (82.4)	<.01	0.62 (0.48-0.80)	0.68 (0.52-0.89)
Rich	465	132 (28.4)	333 (71.6)		Ref	Ref

CI – Confidence interval, RR – Relative risk, Ref – Reference

*Mothers of 7 children died before the beginning of the study.

**Fathers of 3 children died before the beginning of the study.

Almost all women and children reported positive preventive care practices (>95%). However, the reported practices were not associated with antibody positivity (**Table 1**, **Table 2**). In addition, there was no difference in reported CLI, care-seeking, or hospitalization for CLI to antibody positivity status in the preceding three- and six-months period prior to antibody tests (**Table 3**).

**Table 3:** The proportion of women and children who reported symptoms of COVID-Like Illness, sought care and were hospitalized in the preceding six months or three months period before the COVID-19 antibody test by seropositivity status

	Six months prior to sample collection				Three months prior to sample collection			
**Women**	Positive		Negative		Positive		Negative	
	n	%	n	%	n	%	n	%
Total	730	100	1311	100	730	100	1311	100
At least one CLI	96	13.2	178	13.6	56	7.7	108	8.2
At least one sought care due to CLI	1	1.0	6	3.4	0	0.0	5	4.6
At least one hospitalization due to CLI	0	0.0	2	1.1	0	0.0	1	0.9
**Children**	Positive		Negative		Positive		Negative	
	N	%	N	%	N	%	N	%
Total	298	100	1100	100	298	100	1100	100
At least one CLI	54	18.1	216	19.6	36	12.1	146	13.3
At least one sought care due to CLI	19	35.2	63	29.2	14	38.9	33	22.6
At least one hospitalization due to CLI	0	0.0	2	0.9	0	0.0	1	0.7

CLI – COVID-like illness

## DISCUSSION

About one-third of the women and one-fifth of the children in this rural population of Bangladesh were seropositive for SARS-CoV-2 antibodies. The seroprevalence rate increased rapidly beginning May 2021, coinciding with the spread of the Delta variant in Bangladesh [18]. The rates almost doubled for women and tripled for children between March 2021 and July 2021. These increases were almost entirely due to natural infections, as only two women and no child received the COVID vaccine in this population (data not shown). Compared to women and children with the highest household wealth, both women and children from poorer households had a lower risk of infection. Most of the infections were asymptomatic or mild. In addition, the risk of infection among women was higher if she reported chewing tobacco and if her husband was an indoor worker. The risk of infection was higher among children if paternal education was >5 years. Reported preventive care practices in this population were almost universal (>95%), but not associated with the risk of infection.

The inverse relationship between infection and household wealth is difficult to explain. A large body of literature shows that the risk of COVID-19 infection is higher among socioeconomically disadvantaged populations [19-23]. The lower risk among poorer women and children might be due to lower exposure to SARs-Cov-2 or confounding. For example, women who reported that their husbands worked outdoor (eg, farming) were more often from lower wealth households and had a lower risk of COVID-19 infection than women whose husbands worked indoors. Although the adjustment somewhat attenuated the relative risk of infection, the risk among women of lower household wealth was still significantly lower. Likely, we could not capture data on all possible confounders. Although we did not find any correlation between chewing tobacco and household wealth, it doesn't negate the possibility that chewing tobacco was also a confounder.

The lack of effect of public health interventions on COVID-19 infection in our population was presumably due to almost no variation in reported practices. In this rural poor agrarian community, interventions were neither provided nor actively promoted. We did not have data on adherence to or adequacy of the practices. However, the high antibody positivity in this population suggests that adherence and adequacy of the practices might be limited. A recent cross-sectional study from Bangladesh reported that nearly 71% of respondents indicated difficulties adhering to the recommended COVID-19 preventive practices [24]. Lack of interest in using masks, crowded living conditions, inadequate information on the proper application of protective measures, inadequate handwashing and sanitation facilities, and negative influences on family/friends were the main barriers [24]. Several other studies from Bangladesh also identified the unavailability of masks and crowded living conditions as significant barriers to practicing COVID-19 protective behaviours and that the male and rural residents were more likely to face these barriers [25-27]. In a cross-sectional study in the USA, respondents were compliant with straightforward, familiar, and heavily encouraged practices such as handwashing; more burdensome approaches, such as wearing masks, were performed less frequently [28]. These findings are consistent with previous research on preventive care practices for pandemic influenza. Complex interventions such as the use of facemasks in the community require active promotion using multipronged approaches targeting the components of the Health Belief Model, especially perceived susceptibility, potential positive benefits of action, and barriers to actions. We recommend several steps to encourage preventative care adoption. First, clear and consistent messaging from the government and community leaders on the effectiveness of preventive practices may lower the threshold for community acceptance and use. The public should understand that preventive practices affect their risk of contracting COVID-19 and the risk of others becoming infected. Second, compliance with preventive practices should not be burdensome. Masks and hand sanitizer should be readily available and, ideally, freely available.

The vast majority of the cases were asymptomatic or mild in our population. The risk of COVID-19 infection and severity are age- and gender-dependent. Older age is associated with elevated risks of symptomatic illness, severe disease, and death [29]. A study from China reported a higher attack rate in females than males. However, critical illnesses and deaths were significantly lower among females than males [30]. We studied women between 19 and 46 years and children between 35 and 78 months. In addition to younger age, the high coverage of BCG vaccination in our population may be a possible explanation for asymptomatic or mild presentation of COVID -19 [31,32]. BCG is widely used in the developing world, including in Bangladesh. This vaccine prevents infant death from tuberculosis and unrelated infectious agents, especially respiratory tract infections. It has been proposed that these off-target protective effects of the BCG vaccine are mediated by the general long-term boosting of innate immune mechanisms, also termed “trained innate immunity” [33]. Recent studies indicate that COVID-19 incidence and total deaths are strongly associated with the presence or absence of national mandatory BCG vaccination programs [34]. These findings prompted the initiation of several clinical studies to prospectively evaluate the effect of the BCG vaccine on the incidence and severity of COVID-19 [33,35]. A meta analysis evaluated the relationship between BCG vaccination and SARS-CoV-2 infection or COVID-19 disease and found a 40% lower risk of infection in the BCG vaccination group than the control group. However, the evidence that BCG vaccination can reduce the severity of COVID-19 disease is insufficient [36].

Our study had several limitations. First, we studied an existing cohort of women and their children instead of a population-based sample. This cohort was under follow-up in a pregnancy biorepository with important background data and biospecimens available. Second, we collected reported morbidity data but could not confirm the cases for COVID-19 disease because PCR tests were not readily available in this rural population. Third, we collected reported data on preventive care practices and could not collect data on adherence to and the adequacy of the practices. Fourth, we used ROCHE's Elecsys®Anti-SARS-CoV-2 antibody test because this test is highly sensitive and specific. However, the Elecsys®Anti-SARS-CoV-2 antibody test does not differentiate between IGM and IGG. Finally, there were high refusals to blood samples required for serology. However, those who refused the sample were not socioeconomically different from those who provided the blood sample (Table S1 in the **Online Supplementary Document**).

Nonetheless, we provided prospectively collected population-based data, which could contribute to designing feasible strategies against Covid-19 tailored to high-risk groups, particularly in low and middle-income countries such as Bangladesh. The strategies should consider many other consequences of the pandemic, including economic loss, health system stress, and indirect health effects [37-39]. Apart from vaccination, reduced transmission through preventive care practices might be the most feasible strategy for the containment of the pandemic. However, it is not adequate to collect data on reported preventive care practices. Future research should collect more in-depth data to understand the factors related to adoption and adherence to the practices [40].

## Additional material


Online Supplementary Document
