# Supplementary material for: Sero-prevalence and risk factors for Severe Acute Respiratory Syndrome Coronavirus 2 infection in women and children in a rural district of Bangladesh: A cohort study
Source: J Glob Health. 2022 Jul 23;12:05030. doi: 10.7189/jogh.12.05030 (PMC9304923; doi:10.7189/jogh.12.05030)
Supplement: Online Supplementary Document [file jogh-12-05030-s001.pdf]

Table S1. Selected characteristics of women and households by blood sample collection status

Selected Characteristics	Total	Consented for sample	Refused	P-value
	N=2559	n (%)	n (%)	
**Women age in years**				
<30	1723	1361 (79.0)	362 (21.0)	
≥30	836	680 (81.3)	156 (18.7)	0.17
**Women education**				
0-5 years	1116	898 (80.5)	218 (19.5)	
>5 years	1443	1143 (79.2)	300 (20.8)	0.43
**Women occupation**				
Housewife	2524	2012 (79.7)	512 (20.3)	
Working/employed	35	29 (82.9)	6 (17.1)	0.65
**Husband's education***				
0-5 years	1729	1395 (80.7)	334 (19.3)	
>5 years	823	640 (77.8)	183 (22.2)	0.09
**Husband's occupation***				
Govt/private/self-employed (possibly in-door)	936	744 (79.5)	192 (20.5)	
Daily wage/farming/other/does not work (possibly out-door)	1616	1291 (79.9)	325 (20.1)	0.81
**Household Wealth Index (tertiles)**				
Poor	954	774 (81.1)	180 (18.9)	
Middle	797	634 (79.5)	163 (20.5)	0.34
Rich	808	633 (78.3)	175 (21.7)	

* Husbands of 7 women died before the beginning of the study.
